# Retrospective Cohort Analysis of Aspirin Use and Venous Thromboembolism in Patients with Pancreatic Cancer and an Indwelling Central Venous Catheter

**DOI:** 10.1055/s-0042-1747685

**Published:** 2022-08-04

**Authors:** Richard King, Jordan Schaefer, Vaibhav Sahai, Kent A. Griffith, Suman L. Sood

**Affiliations:** 1 Department of Internal Medicine, Division of Hematology and Medical Oncology, University of Michigan Rogel Cancer Center, Ann Arbor, Michigan, United States; 2 Comprehensive Cancer Center Biostatistics Unit, University of Michigan, Ann Arbor, Michigan, United States

**Keywords:** antiplatelet agent, intravascular devices, cancer, venous thrombosis, prevention

## Abstract

**Background** Patients with pancreatic cancer are at high risk of developing venous thromboembolism (VTE). It is unknown if aspirin reduces the risk of VTE in this setting.

**Objectives** We sought to determine whether there is an association between aspirin use and VTE risk in patients with pancreatic cancer receiving chemotherapy with a central venous catheter (CVC).

**Patients/Methods** We conducted a single-center, retrospective cohort study of adult patients diagnosed with pancreatic cancer and treated with chemotherapy using a CVC. Subjects were excluded if they were on anticoagulation at the time of CVC placement. The probability of VTE was analyzed using a time-to-event analysis framework for the development of VTE using the product-limit method of Kaplan and Meier (univariate) and adjusting for important confounding covariates using Cox proportional hazards regression (cause-specific hazard) and again using Fine and Gray regression (subdistributional hazard) with death prior to VTE considered a competing event.

**Results** The final analysis included 314 cases (125 with any aspirin use and 189 without). Patients with any aspirin use had fewer VTE events (34.4%) compared with those without aspirin use (42.3%; *p* = 0.021) by log-rank test and after adjustment for multiple covariates using a Cox proportional hazards model (hazard ratio [HR] = 0.60; 95% confidence interval [CI]: 0.40–0.92; *p* = 0.019). Using Fine and Gray regression to account for death as a competing event, the effect of aspirin remained in the direction of benefit, but was not statistically significant (HR = 0.70; 95% CI: 0.47–1.05, *p* = 0.083). Higher body mass index, active smoking, and metastatic stage of cancer were associated with VTE events in the Cox proportional hazards model. Rates of major bleeding or clinically relevant minor bleeding were similar between treatment groups.

**Conclusions** Aspirin may reduce the risk of VTE in patients with pancreatic cancer with a CVC. We did not observe a significant increase in the rates of major bleeding or clinically relevant nonmajor bleeding.

## Introduction

Patients with pancreatic cancer are at high risk of developing venous thromboembolism (VTE), with an overall incidence of 10 to 23% for patients with advanced or metastatic pancreatic cancer on chemotherapy. 1 2 Outcomes are poor for patients who develop cancer-associated thrombosis (CT) 3 4 ; CT within the first 6 weeks of chemotherapy has been associated with higher risk of death. 5 CT may result in increased morbidity, reduced patient quality of life, and may delay cancer directed therapy.

Many patients with pancreatic cancer have a central venous catheter (CVC) placed to facilitate the administration of chemotherapy, which is an additional risk factor for VTE. Low molecular weight heparin has been previously shown to be effective at preventing VTE in pancreatic cancer. 1 2 In addition, rivaroxaban 6 and apixaban 7 have each been shown to reduce rates of VTE in outpatients with cancer when compared with placebo in randomized trials. A subgroup analysis of patients with pancreatic cancer in the CASSINI trial showed a benefit for rivaroxaban versus placebo. 8

Despite these data, routine pharmacologic VTE prophylaxis in this population remains controversial, with bleeding risk and drug interactions being considerations, and has not been widely implemented. Apart from anticoagulants, less is known about the relative risk and potential benefit of antiplatelet therapy with aspirin for the prevention of VTE in patients with pancreatic cancer. Given that aspirin is a low-cost and generally well-tolerated medication with few drug interactions, it could be an appealing option for VTE prophylaxis for some patients. Aspirin has previously been shown to reduce the risk of recurrent VTE after completion of therapeutic anticoagulation for the treatment of VTE, but the analysis did not specifically examine patients with cancer. 9 Aspirin has been increasingly used for VTE prophylaxis in the postoperative period after total hip or knee replacement, 10 another setting where bleeding risk has been expressed as a concern. In this setting, aspirin has shown similar efficacy to prophylactic anticoagulation in a recent clinical trial 11 and in a recent meta-analysis of randomized controlled trials, 12 and is an option in the American College of Chest Physician guidelines. 13 In contrast, aspirin has not been well studied for the primary prevention of VTE in the setting of solid-tumor malignancies. Major guidelines do not provide specific recommendations regarding antiplatelet therapy for VTE prophylaxis in patients with cancer, other than in the setting of immunomodulatory drug use in multiple myeloma, likely due to the lack of data. 14 15 16 17

At our institution, some of our gastrointestinal oncology providers advocate prophylactic aspirin use and some do not. The objective of this study is to determine whether the use of aspirin is associated with a reduced risk of VTE and CVC-associated thromboembolism in patients with pancreatic cancer. Additionally, we sought to determine the impact of aspirin on the safety outcome of bleeding.

## Methods

We conducted an Institutional Review Board-approved, single-center, retrospective cohort study of adult patients diagnosed with pancreatic cancer (adenocarcinoma, adenosquamous, or acinar histology) from January 2012 to February 2018. Included patients were treated with intravenous chemotherapy, had undergone CVC catheter placement, and had follow-up data for at least 30 days after CVC placement. The day of the CVC placement was the index date; follow-up continued until a VTE, death, or last clinic visit in the electronic medical record. Subjects were excluded if they were on any dose of anticoagulation at the time of CVC placement. The use of aspirin was assessed on review of electronic medical record notes and medication list at the time of CVC placement. This medication list is established by a detailed medication reconciliation process completed by a medical assistant and then verified by a physician.

The primary outcome was VTE during the follow-up period. Determination of VTE was based on review of existing Doppler vascular ultrasound and/or cross-sectional imaging radiology reports in the electronic medical record. Tumor thrombus or vessel occlusion without thrombosis (such as external mechanical vascular compression by tumor) as noted in the radiology report was not considered VTE.

Our secondary outcome was the percentage of subjects who experienced any bleeding events. Bleeding episodes were abstracted from the chart and categorized as major, clinically relevant nonmajor, or nonclinically relevant nonmajor as defined by the International Society on Thrombosis and Haemostasis (ISTH). 18 In the ISTH consensus document major bleeding was defined as “having a symptomatic presentation and at least one of the following criteria: fatal bleeding; and/or bleeding in a critical area or organ, such as intracranial, intraspinal, intraocular, retroperitoneal, intra-articular or pericardial, or intramuscular with compartment syndrome; and/or bleeding causing a fall in hemoglobin level of 20 g/L or more, or leading to transfusion of two or more units of whole blood or red cells.” 18 Clinically relevant nonmajor bleeding was defined as “any sign or symptom of hemorrhage (e.g., more bleeding than would be expected for a clinical circumstance, including bleeding found by imaging alone) that does not fit the criteria for the ISTH definition of major bleeding but does meet at least one of the following criteria: requiring medical intervention by a healthcare professional; leading to hospitalization or increased level of care; prompting a face to face (i.e., not just a telephone or electronic communication) evaluation.” 18 We defined nonclinically relevant, nonmajor bleeding as any bleeding that did not meet criteria for major bleeding or clinically relevant nonmajor bleeding.

For our primary outcome, the probability of VTE was analyzed using a time-to-event analysis framework for the development of VTE using the product-limit method of Kaplan and Meier (univariate, cause-specific hazard) and after considering death prior to VTE as a competing event (univariate, sub-distributional hazard) and to adjust for important confounding covariates using Cox proportional hazards regression (cause-specific hazard) and again when considering death prior to VTE as a competing event using Fine and Gray regression (sub-distributional hazard). In addition, we prespecified an analysis of the probability of VTE at 6 months from the time of CVC placement. Covariates included patient age, sex, body mass index (BMI), history of VTE, current smoking status, Charlson comorbidity index, 19 Eastern Cooperative Oncology Group performance status (0 to 1 versus 2 to 3), Khorana score, 20 and cancer stage at diagnosis, as abstracted from chart review at the time of first outpatient medical oncology clinic visit. These variables were selected based on importance for VTE and bleeding risk as judged by the study team. We defined the follow-up time to be from the date of CVC placement until the occurrence of VTE, loss-to-follow-up, patient death, or end of the study period (the last clinic visit prior to the start date of data abstraction). Death prior to VTE was considered a competing event for such analyses. The proportional hazards assumption for each modeled covariate in the cause-specific hazard models was assessed using the empirical score process of Lin et al. 21 For our secondary outcome of bleeding, we calculated the percentage of subjects who experienced any bleeding event. Within the secondary outcome, subjects were censored at the time of the first bleeding event, and the clinical severity designated based on that bleeding event.

## Results

**Fig. 1:**
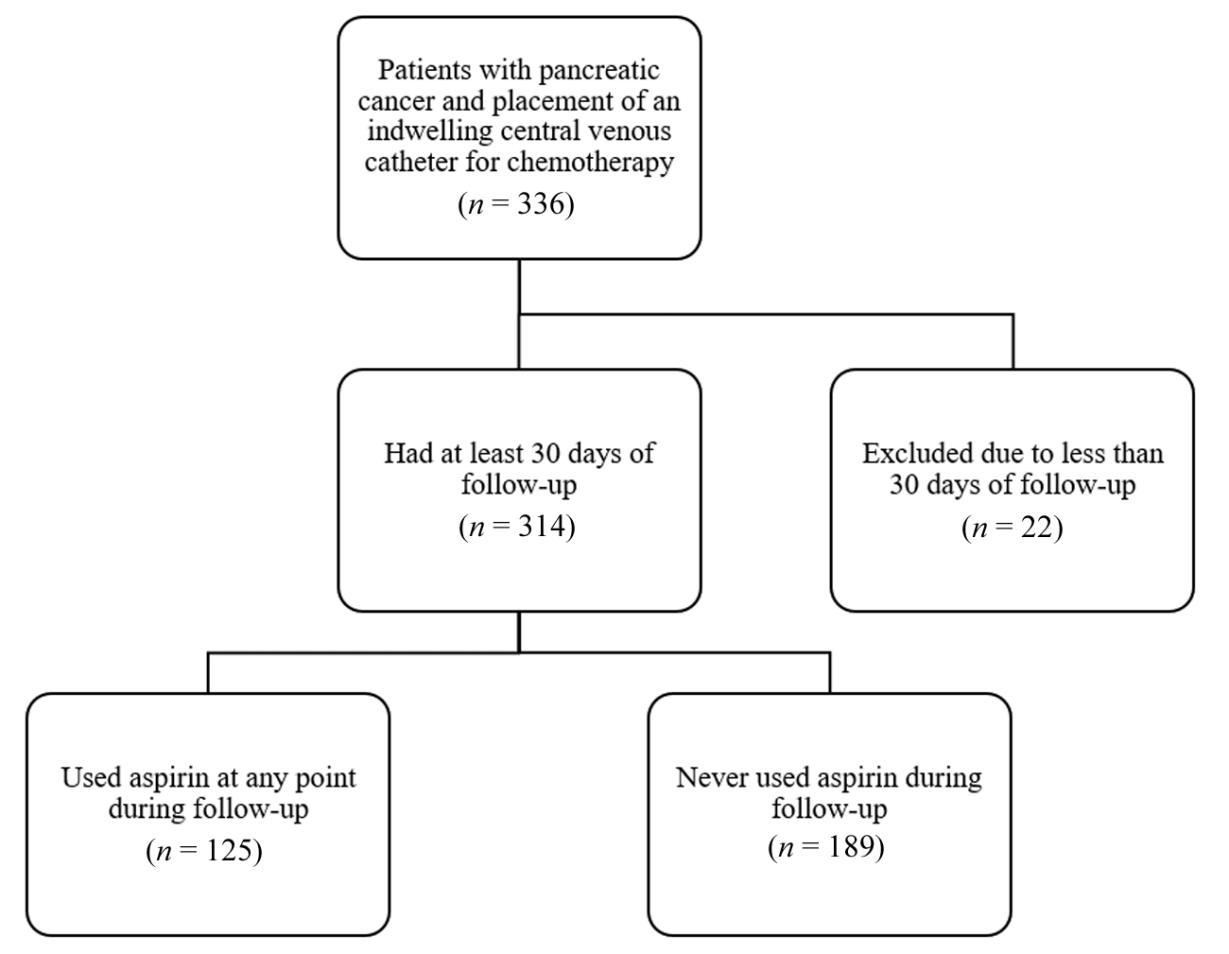
Consolidated standard of reporting trial (CONSORT) flow diagram outlining selection and allocation of cases.

A total of 336 patients (46.7% female) with pancreatic cancer and a median age of 65 years (range, 35 to 89) underwent CVC placement and received chemotherapy. There were 22 patients (8 with any aspirin use; 14 without) excluded due to less than 30 days of follow-up after CVC placement. No patients were started on prophylactic anticoagulation during the follow-up period. Of the remaining 314 patients, 125 reported current use of aspirin while 189 did not ( Fig. 1 ). Of the 125 cases with aspirin use on their medication list, 102 had aspirin listed for at least the first 30 days after line placement, 7 had aspirin listed at the time of line placement but reported stopping aspirin within 30 days after line placement, and 16 started aspirin at a later time. Table 1 shows that the group of patients with any aspirin use after CVC placement were older (67.6 vs. 62.2 years; *p* < 0.01), with a lower proportion of females (36.8 vs. 53.4%; *p* = 0.01), higher Charlson comorbidity index (6.2 vs. 5.7; *p* = 0.04), and more patients had resectable pancreatic cancer at diagnosis (28.0 vs. 15.3%, *p* = 0.01). These confounders were all adjusted for in the analysis.

**Table 1:** Characteristics of patients with pancreatic cancer by aspirin use

	Aspirin	No aspirin	*p* -Value
	*n* = 125 (39.8%)	*n* = 189 (60.2%)	
Age (y)	67.6 ± 9.2	62.2 ± 9.6	<0.01
Race (% White/Caucasian)	88.8%	85.7%	0.43
Sex (% female)	36.8%	53.4%	<0.01
BMI (kg/m ^2^ )	28.5 ± 5.0	27.4 ± 6.0	0.12
History of venous thromboembolism	4.8%	5.3%	0.85
Active smoking	12.9%	16.5%	0.38
Charlson comorbidity index	6.2 ± 2.2	5.7 ± 2.3	0.04
ECOG Performance Status			0.054
0	20.0%	15.3%	
1	51.2%	63.5%	
2	20.0%	12.2%	
3	3.2%	1.1%	
4	0.0%	0.0%	
Missing data	5.6%	7.9%	
Pre-chemotherapy laboratories			
WBC (10^3 cells/µL)	7.9 ± 2.5	8.2 ± 3.2	0.42
Hemoglobin (g/dL)	12.7 ± 1.6	12.7 ± 1.6	0.72
Platelets (10^3 cells/µL)	285 ± 121	278 ± 109	0.58
Serum creatinine (g/dL)	0.85 ± 0.3	0.79 ± 0.3	0.08
Khorana score			0.52
2	58.4%	60.9%	
3	30.4%	29.1%	
4	10.4%	7.4%	
5	0.8%	2.7%	
Stage at diagnosis of cancer			0.013
Resectable	28.0%	15.3%	
Borderline resectable	24.0%	19.0%	
Locally advanced/unresectable	20.0%	27.0%	
Metastatic	28.0%	38.6%	
Follow-up time, months	14.7 ± 14.5	11.1 ± 11.4	0.015

Abbreviations: BMI, body mass index; ECOG, Eastern Cooperative Oncology Group; WBC, white blood cell count.

Plus–minus values are means ± standard deviation.

**Fig. 2:**
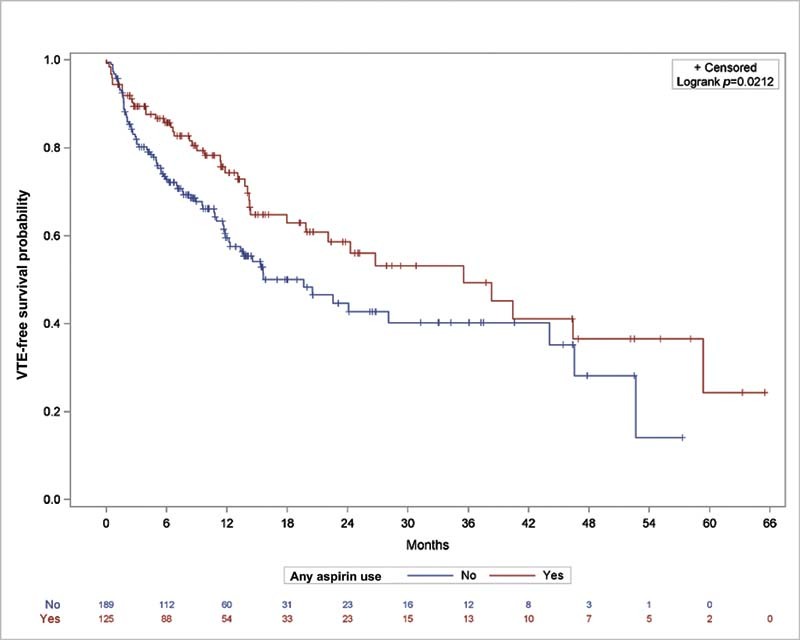
Time-to-VTE event analysis for patients with aspirin (red line) or without aspirin (blue line) use. The number of patients at risk is indicated along the bottom. VTE, venous thromboembolism.

The median follow-up time in the entire population was 8.7 months. Overall, fewer patients (34.4%) with any aspirin use experienced a VTE event during follow-up compared with those without aspirin use (42.3%, *p* = 0.02 by log-rank test for the unadjusted comparison) ( Fig. 2 ). At 6 months after CVC placement, the product-limit estimate, which reflects the probability of being VTE-free, was 85.7% (95% confidence interval [CI]: 78.0%–90.9%) for those with aspirin use and 72.8% (95% CI: 64.8–78.2%) for those without any aspirin use. The estimates were similar when death was considered a competing event: 86.2% (95% CI: 79.5–91.6%) and 73.5% (95% CI: 66.9–79.8%), respectively. Accordingly, the median VTE-free survival for patients with any aspirin use was 1,065 days (95% CI: 595–1,780 days), while it was 589 days (95% CI: 366–1,322 days) for those without any aspirin use.

**Fig. 3:**
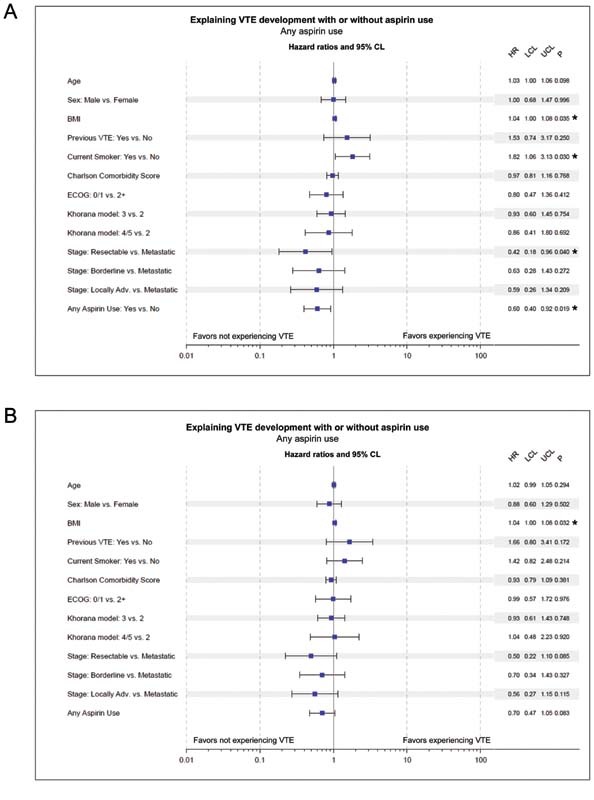
( **A** ) Factors explaining development of VTE after adjustment for important potential confounding covariates, with a Cox proportional hazards model (cause-specific hazard). ( **B** ) Factors explaining development of VTE using Fine and Gray regression (subdistributional hazard) with death prior to VTE considered a competing event. BMI, body mass index; VTE, venous thromboembolism; ECOG, Eastern Cooperative Oncology Group; PS, performance status; HR, hazard ratio; LCL, lower confidence limit; UCL, upper confidence limit. * indicates *p* -value < 0.05.

The difference in rate of VTE events in patients with any aspirin use remained statistically significant after adjustment for important potential confounding covariates, with a Cox proportional hazards model suggesting the aspirin use is protective (HR = 0.60; 95% CI: 0.40–0.92, *p* = 0.019) ( Fig. 3A ). In this model, patients with a higher BMI, active smoking, and metastatic stage of cancer (versus resectable stage) were at significantly higher risk of developing VTE ( Fig. 3 ). Khorana score did not add additional predictive value in this model. The assumption of proportional hazards appeared to be met for all covariates.

The difference in rate of VTE events in patients with any aspirin use remained similar but was not statistically significant after adjustment for important potential confounding covariates when using Fine and Gray regression with death considered as a competing event (hazard ratio [HR] = 0.70; 95% CI: 0.47–1.05, *p* = 0.083). The HR estimates for VTE development for the other covariates for the subdistributional hazard ( Fig. 3B ) were similar to the cause-specific hazard ( Fig. 3A ).

Table 2 details the sites of VTE for patients in this study. Numerically, only one patient with any aspirin use experienced a catheter-related VTE compared with nine patients in the no aspirin-use group ( *p* = 0.056). Isolated lower extremity deep vein thrombosis (DVT) occurred in 8.8% of patients with aspirin use compared with 4.2% of patients without any aspirin use ( *p* = 0.10), while pulmonary embolism (without other known VTE sites) occurred in 8.8% of those who had any aspirin use versus 15.3% of patients without any aspirin use ( *p* = 0.12). Incidentally discovered pulmonary embolism was less frequent with any aspirin use (4.8%) compared with those without aspirin use (13.2%) ( *p* = 0.02). Overall, 42 of the 123 VTE events (34.1%) involved the splanchnic vessels, inferior vena cava, or renal veins. Of these 42 patients, only 18 (43%) were started on anticoagulation, whereas 75 of the 81 patients (93%) with any other site(s) of VTE received treatment with anticoagulation.

**Table 2:** VTE sites and outcomes for patients with pancreatic cancer with or without any aspirin use

	Any aspirin	No aspirin	Total
	*n* = 125 (39.8%)	*n* = 189 (60.2%)	*n* = 314
**VTE**	**43 (34.4%)**	**80 (42.3%)**	**123 (39.1%)**
Catheter-associated	1 (0.8%)	9 (4.7%)	10 (3.2%)
Upper extremity (noncatheter associated)	2 (1.6%)	0	2 (0.6%)
Lower extremity	11 (8.8%)	8 (4.2%)	19 (6.1%)
Proximal	8 (6.4%)	5 (2.6%)	13 (4.1%)
Pulmonary embolism	11 (8.8%)	29 (15.3%)	40 (12.7%)
Incidental a	6 (4.8%)	25 (13.2%)	31 (9.9%)
Subsegmental	3 (2.4%)	8 (4.2%)	11 (3.5%)
Intra-abdominal	14 (11.2%)	28 (14.8%)	42 (13.4%)
Multiple sites	5 (4.0%)	4 (2.1%)	9 (2.9%)
Splenic vein	1 (0.8%)	6 (3.2%)	7 (2.2%)
Portal vein	5 (4.0%)	10 (5.3%)	15 (4.8%)
SMV	3 (2.4%)	4 (2.1%)	7 (2.2%)
Hepatic vein	0	2 (1.1%)	2 (0.6%)
IVC/renal vein thrombosis	0	2 (1.1%)	2 (0.6%)
Multiple systemic sites	2 (1.6%)	6 (3.2%)	8 (2.5%)
Isolated superficial venous thrombosis	2 (1.6%)	0	2 (0.6%)
VTE concurrent with active chemotherapy a	21 (16.8%)	53 (28.0%)	74 (23.6%)
**Stage of cancer at time of VTE**			
Resectable	2/43 (4.7%)	5/80 (6.3%)	7/123 (5.7%)
Borderline resectable	4/43 (9.3%)	8/80 (10%)	12/123 (9.8%)
Locally advanced	9/43 (20.9%)	14/80 (17.5%)	23/123 (18.7%)
Metastatic or recurrent	28/43 (65.1%)	53/80 (66.3%)	81/123 (65.9%)
**VTE cases where anticoagulant was started**	33/43 (76.7%)	60/80 (75%)	93/123 (75.6%)
LMWH	31/33 (93.9%)	47/60 (78.3%)	78/93 (83.9%)
Warfarin	0	0	0
DOAC	2/33 (4.7%)	13/60 (21.7%)	15/93 (16.1%)
**Ischemic stroke or systemic embolism**	**2 (1.6%)**	**0**	**2 (0.6%)**
**Bleeding**			
Major	17 (13.6%)	21 (11.1%)	38 (12.1%)
Clinically relevant nonmajor	9 (7.2%)	10 (5.3%)	19 (6.1%)
Nonclinically relevant nonmajor a	25 (20.0%)	18 (9.5%)	43 (13.7%)
**Deaths during evaluable period**	**69 (55.2%)**	**103 (54.5%)**	**172 (54.8%)**

Abbreviations: DOAC, direct oral anticoagulant; IVC, inferior vena cava; LMWH, low-molecular weight heparin; VTE, venous thromboembolism.

Percentages are out of the total number of subjects in the column unless specifically noted by the denominator shown.

a *p* < 0.05 by Fisher's exact test (two-tailed). No other differences between the groups were significantly different at an α-level of 0.05 using Fisher's exact test (two-tailed).

Major bleeding occurred in 11.1% of subjects without aspirin versus 13.6% with aspirin ( *p* = 0.60) ( Table 2 ). The occurrence of clinically relevant nonmajor bleeding was also similar between subjects without aspirin use (5.3%) and with aspirin use (7.2%, *p* = 0.48). Aspirin was associated with a higher proportion of subjects experiencing nonclinically relevant nonmajor bleeding (20.0%) versus 9.5% for subjects without aspirin ( *p* = 0.01).

## Discussion

Patients with pancreatic cancer receiving chemotherapy are at increased risk of both VTE and bleeding. It is crucial to develop strategies to reduce the risk of CT without an unacceptable increase in bleeding. In this retrospective cohort study of this high-risk population, we found a significant reduction in the rate of VTE for patients who took aspirin at any point after placement of a CVC compared with those not on aspirin in our cause-specific model. When accounting for death as a competing event for the development of VTE, the protective effect of aspirin was not statistically significant, but remained in the same direction of potential benefit for aspirin use. Major or clinically relevant nonmajor bleeding occurred in a similar proportion of patients whether taking aspirin or not, but there was a significant increase in minor bleeding associated with aspirin use.

Approximately 39% of patients in our cohort were using aspirin. This is slightly higher than the ∼30% aspirin use anticipated among adults over 40 years old in the general population 22 and is likely due to the more advanced age of the patient population. In addition, some oncologists in our group advocate for aspirin use, and this may influence these numbers. The overall incidence of VTE in this population was 38.8% during a median follow-up of 8.7 months. The high event rate in this study may be due to a significant proportion of patients experiencing splanchnic vein thrombosis, which accounted for 32.5% of events.

Less than half of patients with splanchnic vein thrombosis were started on anticoagulation. The retrospective nature of our study limited our ability to reliably ascertain the presence or absence of symptoms related to splanchnic vein thromboses, especially because this study focused on pancreatic cancer patients, who often experience abdominal symptoms due to the cancer. An improved understanding of the relative risk of anticoagulation (bleeding) compared with the potential for benefit (potentially improved rates of recanalization, reduced risk of intestinal ischemia or portal hypertension, etc.) in this cohort is needed, especially with consideration to their poor overall prognosis. The serial abdominal imaging performed to monitor their cancer also allows close follow-up of their intra-abdominal thrombosis, which may have guided decisions regarding anticoagulation.

Aspirin use for the primary analysis was dichotomized to any aspirin exposure or no aspirin exposure. Given the dynamic use of aspirin in this group with changes potentially relating to bleeding or laboratory abnormalities, this definition seemed appropriate given that we adjusted for other potential confounders. Aspirin being added to a patient's medication list after 30 days may have been a result of more accurate medication reconciliation rather than a patient newly starting the medication; aspirin removal from the medication list seemed most often a change due to clinical parameters (bleeding, thrombocytopenia, etc.).

This study adds to our knowledge of prophylactic agents for CT, showing the relative efficacy and safety of an antiplatelet agent, aspirin, to prevent VTE in high-risk patients with cancer. The low molecular weight heparin dalteparin was shown to reduce all-type VTE from 28 to 12%, 1 and enoxaparin showed a reduction in VTE rate from 15.1 to 6.4% in patients receiving chemotherapy for pancreatic cancer. 2 The CASSINI 6 and AVERT 7 trials showed that rivaroxaban or apixaban, respectively, compared with placebo, reduced the risk of VTE in high-risk patients across different cancer types during the intervention period. A subgroup analysis of patients with pancreatic cancer in the CASSINI trial showed a benefit for rivaroxaban versus placebo (HR for the composite of symptomatic DVT, asymptomatic proximal DVT, any PE and VTE-related death was 0.35 with a 95% CI 0.13–0.97, with no increase in major bleeding events (1.5% with rivaroxaban versus 2.3% with placebo)). 8 In our study, the HR for VTE with aspirin was 0.60 with a 95% CI 0.40 to 0.92. Consistent with our findings, a recent large retrospective analysis of patients 65 or older with cancer reported an association with aspirin use and reduction in VTE. 23 Aspirin may be an attractive alternative option for patients who are not candidates or do not prefer full or prophylactic dose anticoagulation, such as those taking concomitant medications that can lead to significant drug interactions, impaired renal or hepatic function, or with a high bleeding risk. The fact that aspirin is oral and low cost may also be favorable for some patients.

Strengths of the study include the availability of complete staging information, which is often not available in claims data, complete follow-up information with a low number of patients lost to follow-up, and the continued significance of the findings after adjusting for covariates. Limitations of this study include those inherent to a retrospective chart review, including being limited to data abstracted from the medical record, a lack of randomization with a risk of confounding, and reliance on reported aspirin use. Documentation of the indication for aspirin was inconsistent in the medical record, and we were unable to definitively ascertain the primary indication for aspirin as a result. Some patients in the aspirin group may have been taking aspirin for primary vascular prophylaxis rather than VTE prevention. We attempted to minimize bias by adjusting for confounding variables, setting definitions for variables prior to data collection, and using existing imaging reports rather than reinterpreting imaging reports. There were more patients in the aspirin group who had resectable disease, although aspirin use remained protective even after this important covariate was accounted for in the Cox proportional hazards model. Average follow-up time was longer in the aspirin cohort than the no aspirin cohort, which if anything could bias toward increased events in the aspirin cohort, but this longer follow-up time did not result in an increase in VTE or major or clinically relevant bleeding events in the aspirin cohort. The study size was small, and accordingly the study may be underpowered to assess for some outcomes. Included patients generally received all their medical and emergency care through our center. However, it is possible that bleeding or thrombotic events managed at outside facilities were not captured. As our observed bleeding events seem generally consistent with those reported for this population in the medical literature, we do not think this significantly influenced our findings.

## Conclusion

Low-dose aspirin may reduce the risk of VTE in patients with pancreatic cancer undergoing chemotherapy after CVC placement. The protective effect of aspirin use on development of VTE in our primary analysis (Cox proportional hazards regression) was not statistically significant in our secondary analysis (Fine and Gray regression to account for death as a competing event for VTE development), but remained consistent in the direction of benefit with aspirin use. Minor bleeding was increased with aspirin, but we did not observe a significant increase in major bleeding or clinically relevant nonmajor bleeding. Aspirin may prove to be a low-cost, well-tolerated alternative agent for the primary prophylaxis of VTE in patients with pancreatic cancer receiving chemotherapy, especially in patients who are not candidates or interested in anticoagulant prophylaxis. Larger studies are needed to confirm these findings.

## Addendum

R. King contributed to study design, performed data collection and interpretation, contributed to statistical analysis, and wrote the manuscript. J. Schaefer, V. Sahai, and S. Sood contributed to study design, data interpretation, and writing and revising the manuscript. K. Griffith performed the statistical analyses, data interpretation, and provided critical contributions to revising the manuscript.

## Notes

**What Is Known on This Topic?**

- Patients with pancreatic cancer are at high risk of VTE, which is associated with worse outcome.
- Systemic anticoagulation has been shown to reduce rates of VTE for outpatient cancer patients.
- Aspirin reduces the risk of recurrent VTE after treatment with anticoagulation in the general population. It is unclear if aspirin is effective for preventing venous VTE in pancreatic cancer.

**What Does This Paper Add?**

- For patients with pancreatic cancer and undergoing chemotherapy treatment through a CVC, aspirin is associated with a reduced risk of VTE.
- Major or clinically relevant nonmajor bleeding risks are similar with or without aspirin.
- Aspirin could be a low-cost, well-tolerated alternative for outpatient primary VTE prophylaxis in patients with pancreatic cancer. Additional studies are needed to confirm.
